# Disparities in emergency department use between Italians and migrants residing in Rome, Italy: the Rome Dynamic Longitudinal Study from 2005 to 2015

**DOI:** 10.1186/s12889-020-09280-6

**Published:** 2020-10-15

**Authors:** Eleonora Trappolini, Claudia Marino, Nera Agabiti, Cristina Giudici, Marina Davoli, Laura Cacciani

**Affiliations:** 1 Sapienza University of Rome, Rome, Italy; 2 Department of Epidemiology, Lazio Regional Health Service, Rome, Italy

**Keywords:** Emergency department, Healthcare use, Migrant population, Dynamic cohort, Disparities, Great Recession

## Abstract

**Background:**

The Emergency Department (ED) can be considered an indicator of accessibility and quality and can be influenced in period of economic downturns. In the last fifteen years, the number of migrants in Italy has doubled (from 2.4 million in 2005 to 5.2 in 2019, 4.1 and 8.7% of the total population, respectively). However, evidence about migrants’ healthcare use is poor, and no studies focused on the ED utilisation rate during the Great Recession are available. This study aims to analyse trends in all-cause and cause-specific ED utilisation among migrants and Italians residing in Rome, Italy, before and after 2008.

**Methods:**

Longitudinal study based on data from the Municipal Register of Rome linked to the Emergency Department Register from 2005 to 2015. We analysed 2,184,467 individuals, aged 25–64 in each year. We applied a Hurdle model to estimate the propensity to use the ED and to model how often individuals accessed the ED.

**Results:**

Migrants were less likely to be ED users than Italians, except for Africans (RR = 1.46, 95%CI 1.40–1.52) and Latin Americans (RR = 1.04, 95%CI 1.00–1.08) who had higher all-cause utilisation rates than non-migrants. Compared to the pre-2008 period, in the post-2008 we found an increase in the likelihood of being an ED user (OR = 1.34, 95%CI 1.34–1.35), and a decrease in ED utilisation rates (RR = 0.96, 95%CI 0.96–0.97) for the whole population, with differences among migrant subgroups, regardless of cause.

**Conclusions:**

This study shows differences in the ED utilisation between migrants and Italians, and within the migrant population, during the Great Recession. The findings may reflect differentials in the health status, and barriers to access primary and secondary care among migrants. In this regard, health policies and cuts in health spending measures may have played a key role, and interventions to tackle health and access disparities should include policy measures addressing the underlying factors, adopting a Health in All Policies perspective. Further researches focusing on specific groups of migrants, and on the causes and diagnoses related to the ED utilisation, may help to explain the differences observed.

## Background

The migrant population residing in Italy grew exponentially in the last two decades, from 1.3 million in 2001 to more than 5.2 million in 2019 (respectively, 2.3 and 8.7% of the total population). Italian migration flows have diversified over time and are not just linked to a few countries: in 2019 it is possible to count 195 different nationalities [1]. Although the main reason to migrate in Italy was for labour opportunities, indeed most of migrants came from low income countries attracted by a high demand for unskilled jobs, especially in the sectors of construction and domestic services [2], two relevant episodes partially affected the recent Italian migration flows. Firstly, the anti-government protests occurred in the Arab countries, known as the Arab Spring, which began around 2011; secondly, the geopolitical transformations and conflicts in the Middle East and Sub-Saharan Africa, occurred between 2014 and 2015 [3]. As a consequence of those events, many asylum seekers and applicants for international protection arrived in Italy.

Rome, the capital of Italy, hosts a high concentration of migrants, which has increased rapidly, from 3.9% in 2001 to 13.4% of the total resident population in 2019. The plurality of countries of origin reflect different cultures, behaviours, and health needs of the migrant population. Currently, the largest migrant communities settled in Rome are Romanians (24%) and Filipinos (11%) [1], mainly females, attracted by domestic jobs [4]. The share of asylum seekers and holders of international protection out of the total number of migrants residing in Rome was very low at the beginning of the period covered by our study, and increased with the refugees’ emergency, especially from 2013 when the city entered the national reception system [5], reaching a peak of 4% only in 2015 [6].

Nowadays, migration represents a challenge for every European country as the continent shifts towards a multi-ethnic and multi-cultural reality. One of the most worrisome political issue regarding the migration phenomenon, and one of the greatest challenges for host countries is the effective management of migrants’ healthcare needs in terms of equity, access, and appropriateness of services. In 2008, the WHO’s Commission on Social Determinant of Health highlighted the rise of new health inequalities between and within countries due to differences in social class, gender and ethnicity; ethnicity referred in part to unequal healthcare use by migrants and non-migrants [7]. More recently, the persistence of social inequalities in modern welfare states has been shown [8]. The Great Recession, caused by a global economic downturn in the late 2007 in the United States as a consequence of the financial crisis, which deeply affected jobs and savings of families around the world, have further worsened the health conditions of individuals, not least in terms of access to healthcare systems [9–11], with particularly severe effects in European Southern countries [12, 13], due to the conjunction of recession and stark austerity policies. Although it is difficult to produce evidence about the influence of the recession on health [14], some European studies show a recent change in individual behaviours related to the healthcare use [15–20] that could affect the future health status of the populations.

Disparities in health services use may be related to differences in health status, lifestyles, and preparedness to seek health care. Then, there are linguistic, bureaucratic, cultural and organizational barriers, the fear of discrimination, and different factors mainly related to the perception of illness or to what has been called health literacy. Selection hypotheses should also be considered. The *healthy migrant effect*, according to which migrants are more likely to report better health than non-migrants, because only those in good health are prone to migrate; and the *salmon bias* which argues that unhealthy migrants are more likely to return to their home countries [21, 22]. However, this selection effect may be offset by the poor conditions (economic, environment, social and housing) migrants experience in the host country (the *exhausted migrant effect*) [23–25]. Such inequalities are also related to differences in morbidity, self-perceived health, and healthcare use. Healthcare use is particularly difficult to evaluate and to measure. Several factors can facilitate or impede the use of given healthcare services. The first author to study healthcare demand was Andersen in the 1960s. With the Andersen behavioural model, the author specified four macro categories of explanatory variables considered to be the driving factors of individuals’ access to healthcare services. First, he argued that individuals’ use of health services is a function of contextual factors in each country, namely, healthcare organization and the political, economic, and social setting. Second, predisposing factors or characteristics, like demographic attributes, health beliefs. Third, enabling factors, those factors which enable or impede individuals from using healthcare services, as social and financial resources. Fourth, individuals’ need for healthcare, and health needs. These factors are supposed to have a different explanatory power for each healthcare outcome: physician and ambulatory care; hospital and in-patient services; and dental care [26].

The Italian National Health Service (SSN, Law 883/78) is organised on a regional basis. It provides universal coverage free of charge to all citizens and legal migrants for primary healthcare services, inpatient care, and prevention services, with small co-payments for outpatient specialist care visits and some drugs. Private health insurance has a limited role in Italy’s health coverage system. Approximately 10% of the total population have some form of voluntary health insurance, which covers services excluded under SSN essential benefits. Health policies which regulate migrants’ health are to be found in the *D. Lgs. 286/98,* including the *Single text on Immigration* approved in 1998. These provisions guarantee equal rights in terms of health and healthcare use for both Italians and migrants who are legally present in the country, and provide emergency health assistance for irregular migrants, through the Emergency Department, especially for women and children [27]. Furthermore, any irregular migrants can benefit from medical assistance (essential and urgent care) with a special code named *STP - Straniero Temporaneamente Presente* (temporary foreigner), which is valid for one year on the national territory. Even if in our country equity is one of the most important fundamental principle of the SSN, most health policies are developed and implemented by regions, thus, there is an increasing regional heterogeneity in terms of quality and quantity of care offered to citizens [28].

Although differences in the healthcare services use between migrants and non-migrants have been documented in the international literature [29–32], only a few studies analyse migrants’ use of Emergency Departments (hereafter, ED). The handful of studies available provide fragmented and context-depending results [33]. Cross-sectional works found higher ED use among migrants [30, 34–37], especially for non-urgent conditions [38, 39], and with differences among migrant subgroups [40, 41]. Others showed no differences [29, 42], or lower ED use by migrants as compared to non-migrants [30, 43–46].

In the Italian literature, evidence on migrants’ health and their use of health services is poor [47–55], and few studies focus on the ED utilisation rate [56–59]. What is more, as far as we know there are no studies which analyse changes in ED use during the Great Recession.

Our study contributes to the literature about migrants’ healthcare use with a comparative analysis of ED utilisation trends for migrants and Italians residing in the Municipality of Rome. Using a longitudinal approach based on a dynamic population cohort, the aim of this study is twofold. First, ED use is analysed by migrant status in order to explore whether there are differences between the Italian and the migrant population residing in Rome, from 2005 to 2015. Second, the study analyses changes in ED use by comparing the pre-2008 and the post-2008 period, for all causes and for selected causes (cardio-vascular diseases -CVDs, mental disorders, and injuries). We selected these three groups of diagnosis because it has been observed that mortality related to such areas is susceptible to economic shocks [60]. We hypothesise that: 1) Migrants tend to register lower ED use with respect to Italians due to their better health. 2) Over time, migrants’ health status may worsen due to economic downturns, and to changes in lifestyles, with poor living and working conditions in the host country.

## Methods

### Study setting, design and cohort description

The study was set in Rome, the capital of Italy. Rome is situated in the Lazio region in central Italy. The city counts 2.8 million residents, and it is the first Italian city by number of migrants (more than 380,000) [1].

A longitudinal study was conducted, using the Rome Dynamic Longitudinal Study cohort, which is part of the Italian Network of Longitudinal Metropolitan Studies (IN-liMeS). The cohort is based on the Rome Municipal Register, which provides demographic information (sex, birthdate, birthplace, citizenship, date of registration in the Municipality of Rome and date of cancellation from the population register) for all who have been resident in Rome from 1st January 2001 to 31st December 2015 (4,143,462 records). See Caranci et al. (2018) [61] for more detailed information on how the cohort was derived.

We started from the cohort data and we analysed data of residents from 01/01/2005 to 31/12/2015 (3,845,833 records). We selected all residents, who were aged 25 to 64 years in each calendar year. Entry into the study population can take place for immigration or age (≥ 25 years old). Exit, meanwhile, can come about because of emigration, age (≥ 65 years old), death or for the end of the study (2,422,947 records). Afterwards, to obtain information on the ED use by each individual, we linked the cohort data with the Lazio Health Information System on Emergency Care data, which provides information about all ED contacts in the Lazio region, through the Health Information Systems code (an anonymised code) and ED access date, only for Italians and regular migrants. Individuals who did not have a reliable Health Information Systems code were 1.1% (2,397,475 records), thus the final study population (25–64 years-old) is composed of 2,184,467 individuals.

### Study variables

#### Outcome

The outcome variable is the number of ED contacts per subject. In addition to the total ED contacts, we analysed the following specific groups of diagnosis (coded according to the ICD-9-CM): cardiovascular diseases (CVDs, codes 401–445); mental disorders (290–319); and injuries (800–959).

#### Exposures and control variables

The exposure variable is the origin area (migrants vs. Italians). This was measured by citizenship at first entry into the cohort. This study defines as migrant those individuals without Italian citizenship, further distinguishing migrants from High Migratory Pressure Countries (HMPC: Central-Eastern Europe, Africa, Asia -except for Israel and Japan-, and Latin America) and migrants from all the other countries, i.e. Highly Developed Countries (HDC).

Time-period (pre-2008: 01/01/2005–31/12/2008, reference category; post-2008: 01/01/2009–31/12/2015) is considered as an effect modifier, while age (25–34 years, reference category; 35–44; 45–54; 55–64), and gender (males as reference category) are considered as confounders.

### Statistical analysis

In order to explore changes in the use of emergency service, in the first part of the study we investigated and compared the trend and pattern of ED use by origin area (Italy, HMPC and HDC). The utilisation rate is defined as the ratio between the number of ED contacts and the person-time of the resident in Rome *per* 1000, in each year. Direct age-standardized utilisation rates (SUR) were computed using the population residing in the Lazio region on 1st January 2014 as the standard population.

Subsequently, we applied the Hurdle Model (a two-part model) which allows to consider two important characteristics when analysing the healthcare use. The first one is related to the peculiarity of the dependent variable which is represented by a count outcome (in this study, it is the number of times that individuals contact the ED) [62]; and the second is related both to the occurrence of the outcome (i.e., ED use or no ED use) and the frequency of multiple attendances [63]. One-part models, as Poisson or Negative Binomial regression which are usually considered more appropriate techniques with count data [64], assume that events occur independently over time. In this case, the independence assumption implies that the probability of the *n*th visit to the healthcare service is independent of the *(n + 1)*th and *(n-1)*th visits. However, when considering the healthcare use, this is a very restrictive assumption and inconsistent with the idea that such phenomenon commonly exhibits dynamic dependence. Indeed, there might be some form of dependence between successive events, for instance with repeat attendances either for the same or different problem [65]. Conversely, the Hurdle model allows to model and distinguish between the probability that a person has any healthcare use - i.e., ED use or no ED use - and the frequency of use, separately [66]. There is also a third reason why two-part models offer better techniques for the estimation of healthcare use. Poisson and Negative Binomial models often predict a lower proportion of zeros than is observed in the data [67]. In our study, the number of zero-observations is 2,037,216, taking both periods separately (pre-2008 and post 2008); while the Negative Binomial model predicts only 1,244,069 zero-observations.

In this study, in the first step, a logit is run to estimate the propensity for being an ED user (zero-hurdle part), using the full study population. The second part (count-part) is represented by a zero-truncated Negative Binomial for handling overdispersed count data. The count part estimates the average number of ED contacts on the subpopulation of people who have ED experience. In addition, by separating out the decision for any ED contact (zero versus non-zero) from the frequency of use (how much if non-zero), it may be possible to assess whether migrant status has its effect largely through the contact decision or through the frequency decision in medical care.

We modelled the interaction between the origin area and the time-period. We considered two-sided *p*-values less than 0.05 as being statistically significant.

The software SAS 9.4 was used for data management, while all calculations were performed using the software R.

## Results

The total study population is composed of 2,184,467 individuals with an average follow-up time of 7.84 years. As mentioned in the methods section, we did not include individuals who did not have reliable information about the Health Information System code (1.1%).^1^

Respectively in the pre-2008 and in the post-2008 time-period, the average age of individuals was quite similar (around 44 years-old), the proportion of women was higher than men, and 11.5% (out of 1,737,105 individuals) and 18.1% (out of 2,035,479) were migrants. Most of migrants came from Central-Eastern Europe, followed by Asia, Africa, Latin America and HDC (Table 1).

**Table 1:** Demographic characteristics of the population residing in Rome aged 25–64 years, by time-period

	Pre-2008		Post-2008	
Total	1,737,105	100	2,035,479	100
**Mean age** ***(SD)***	44.1 *(10.8)*		44.4 *(10.7)*	
	N	%	N	%
**Gender**				
M	837,517	48.2	994,205	48.8
W	899,588	51.8	1,041,274	51.2
**Origin area**				
Italian	1,537,361	88.5	1,667,303	81.9
HDC^a^	20,554	1.2	27,702	1.4
HMPC^b^	179,190	10.3	340,362	16.7
*of which*				
Africa	26,087	13.1	50,488	13.7
Latin-America	24,043	12.0	38,861	10.6
Asia	53,308	26.7	112,916	30.6
Central-Eastern Europe	75,752	37.9	13,209	37.6

^a^ HDC: Highly Developed Countries; ^b^ HMPC: High Migratory Pressure Countries

From 2005 to 2015, 4,291,795 ED contacts were registered. In the post-2008, a decrease in the standardized utilisation rates was detected compared to the pre-2008 for all-cause ED contacts (251‰ vs. 271‰) and injuries (83‰ vs. 93‰). No changes occurred for cardiovascular diseases and mental disorders (6‰ and 7‰ for the two time-periods); and some differences by origin area were found (Table 2).

**Table 2:** Absolute numbers and standardized utilisation rates of the Emergency Department contacts

	Pre-2008											
	All-cause			CVDs			Mental disorders			Injuries		
	N^**a**^	%	SUR^**b**^ × 1000	N	%	SUR × 1000	N	%	SUR ×1000	N	%	SUR ×1000
Total	1,637,551	100	271	38,620	2.4	6	38,826	2.4	7	551,798	33.7	93
**Origin country**												
Italy	1,510,893	92.3	279	37,005	95.8	6	37,164	95.7	7	520,153	94.3	99
HDC^c^	6078	0.4	87	72	0.2	1	89	0.2	1	1823	0.3	28
HMPC^d^	120,580	7.4	207	1543	4.0	3	1573	4.1	3	29,822	5.4	
* of which*												
Africa	21,143	1.3	236	231	0.6	3	274	0.7	3	4804	0.9	51
Asia	28,733	1.8	155	584	1.5	3	276	0.7	1	6416	1.2	34
Latin-America	20,667	1.3	246	133	0.3	2	249	0.6	3	4995	0.9	57
Central-Eastern Europe	50,037	3.1	229	595	1.5	4	774	2.0	3	13,607	2.5	57
	Post-2008											
	All-cause			CVDs			Mental disorders			Injuries		
	N	%	SUR ×1000	N	%	SUR × 1000	N	%	SUR ×1000	N	%	SUR ×1000
Total	2,654,244	100	251	72,472	2.7	6	64,704	2.4	7	786,208	29.6	83
**Origin country**												
Italy	2,311,510	87.1	261	66,348	91.5	6	59,560	92.0	7	710,904	90.4	85
HDC	11,311	0.4	93	207	0.3	2	194	0.3	2	3006	0.4	28
HMPC	331,423	12.5	208	5917	8.2	4	4950	7.7	3	72,298	9.2	44
* of which*												
Africa	50,321	1.9	243	793	1.1	4	736	1.1	3	9844	1.3	43
Asia	80,809	3.0	161	2063	2.8	4	726	1.1	1	15,462	2.0	30
Latin-America	48,332	1.8	251	476	0.7	3	604	0.9	3	10,788	1.4	57
Central-Eastern Europe	151,961	5.7	222	2585	3.6	4	2884	4.5	4	36,204	4.6	52

^a^ N: absolute numbers; ^b^ SUR: Standardized Utilisation Rates; ^c^ HDC: Highly Developed Countries; ^d^ High Migratory Pressure Countries

**Fig. 1:**
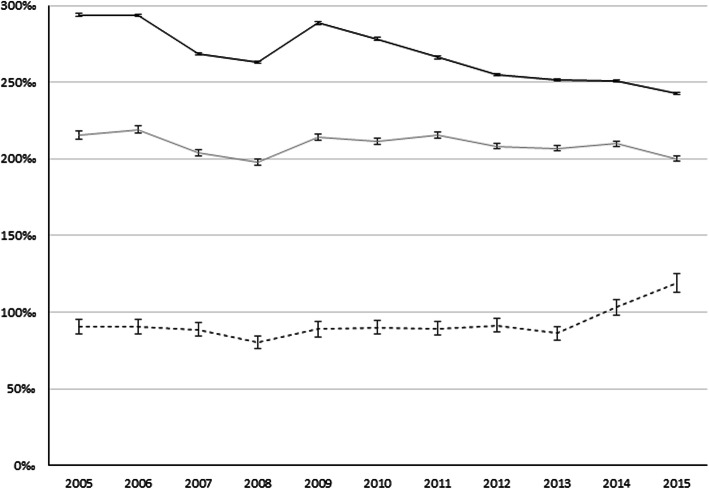
Trends of all-cause standardized ED utilisation rates by migrant status (SURx1000). Legend:

Looking at trends of all-cause ED utilisation rates by migrant status (Fig. 1), in the post-2008 period, an overall decrease of all-cause standardized utilisation rates was registered among both Italians and migrants from HMPC. The trend was only temporarily altered in 2009 (289‰ among Italians and 214‰ among migrants), when the GR set-up, and then decreased again in the following years, though with a lower slope. A slight increase was, instead, observed among migrants from HDC.

**Fig. 2:**
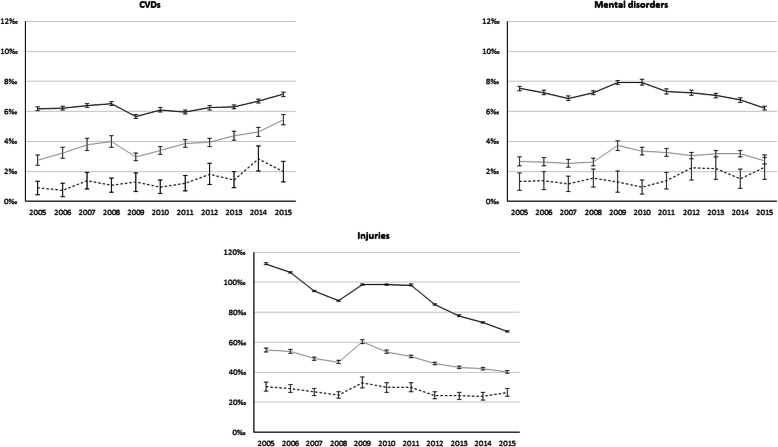
Trends of standardized ED utilisation rates by migrant status and selected causes (SUR× 1000). Legend:

Different patterns were detected for the three selected causes (Fig. 2). For cardio-vascular causes, an increase of standardized utilisation rates in the post-2008 period was observed both for Italians and migrants, while for the other causes there was a decrease. Only mental disorders and injuries seemed to contribute to the overall decrease in all-cause SUR, and to the spike in contacts observed for all causes in 2009 (Fig. 1).

### All-cause emergency department contacts

Tables 3 and 4 display Hurdle Model results for ED use according to the origin area, while results distinguishing between Italians, HMPC and HDC are commented below. Regression results for adjusting factors and interactions used in the model are presented in Tables 3A and 4A in the Additional file.

**Table 3:** Hurdle model: all-cause ED contacts by origin area and time-period, 2005–2015

		Zero-part model			Count-part model	
	N	OR	95% CI	N	RR	95% CI
**Origin area**						
Italy^a^						
HDC^b^	48,256	0.23	(0.22–0.24)	8893	0.65	(0.61–0.70)
Africa	76,575	0.52	(0.50–0.53)	24,219	1.46	(1.40–1.52)
Latin-America	62,904	0.72	(0.70–0.73)	26,586	1.04	(1.00–1.08)
Asia	62,904	0.43	(0.43–0.44)	50,093	0.80	(0.78–0.83)
Central-Eastern Europe	213,961	0.55	(0.54–0.55)	83,619	0.96	(0.93–0.98)
**Time-period**						
Pre-2008^a^						
Post-2008	2,035,479	1.34	(1.34–1.35)	989,346	0.96	(0.96–0.97)
* N observations*	*3,772,584*			*1,735,368*		

^a^ Reference category

^b^ Highly Developed Countries

Adjusted for: gender and age.

Zero-part model reports odds ratios for the outcome variable indicating persons without (Y = 0) or with Emergency Department experience (Y = 1, where all values larger than 0 are censored, which means, are fixed at 1), while the Count-part model, which reports rate ratios, models the number of Emergency Department experiences for those with Emergency Department experiences (for those with Y > 0)

**Table 4:** Hurdle Model: cardiovascular diseases, mental disorders, injuries ED contacts by origin area and time-period, 2005–2015

		CVDs					Mental disorders					Injuries				
	*N* *Zero-part model*	Zero-part model			Count-part model		Zero-part model			Count-part model		Zero-part model			Count-part model	
		OR	95% CI	*N*	RR	95% CI	OR	95% CI	*N*	RR	95% CI	OR	95% CI	*N*	RR	95% CI
**Origin area**																
Italy^a^																
HDC^b^	48,256	0.17	(0.14–0.22)	232	0.77	(0.37–1.62)	0.22	(0.17–0.28)	183	0.71	(0.39–1.29)	0.25	(0.23–0.26)	3790	0.69	(0.62–0.77)
Africa	76,575	0.47	(0.40–0.54)	773	1.09	(0.75–1.58)	0.47	(0.41–0.54)	640	0.78	(0.56–1.09)	0.42	(0.40–0.43)	9723	0.89	(0.84–0.95)
Latin-America	62,904	0.35	(0.29–0.42)	486	0.97	(0.58–1.62)	0.54	(0.47–0.62)	692	0.41	(0.28–0.61)	0.58	(0.56–0.60)	10,931	0.85	(0.80–0.90)
Asia	62,904	0.59	(0.54–0.65)	2096	1.05	(0.83–1.34)	0.24	(0.21–0.28)	734	0.63	(0.45–0.88)	0.31	(0.30–0.32)	16,942	0.61	(0.58–0.65)
Central- Eastern Europe	213,961	0.49	(0.45–0.54)	2466	1.19	(0.94–1.51)	0.49	(0.45–0.53)	2566	0.62	(0.50–0.77)	0.47	(0.46–0.48)	34,543	0.85	(0.82–0.89)
**Time-period**																
Pre-2008^a^																
Post-2008	2,035,479	1.60	(1.57–1.62)	54,345	0.88	(0.85–0.91)	1.31	(1.28–1.33)	34,599	0.91	(0.88–0.95)	1.21	(1.20–1.22)	501,157	0.82	(0.81–0.83)
*N observations*	*3,772,584*			*84,610*					*57,820*					*882,930*		

^a^ Reference category

^b^ Highly Developed Countries

Adjusted for: gender and age.

Zero-part model reports odds ratios for the outcome variable indicating persons without (Y = 0) or with Emergency Department experience (Y = 1, where all values larger than 0 are censored, which means, are fixed at 1), while the Count-part model, which reports rate ratios, models the number of Emergency Department experiences for those with Emergency Department experiences (for those with Y > 0)

From 2005 to 2015, the zero-part model displays that all migrant subgroups were less likely to be ED users than Italians. However, the count-part model indicates that among the subsample of individuals who had ED experience, Africans (RR = 1.46) and Latin Americans (RR = 1.04) had higher ED utilisation rates than Italians, while other groups registered lower rates.

Looking at the relation between ED utilisation and time-period, compared to the pre-2008, in the post-2008 time-period the probability that an individual has any ED use was higher (OR = 1.34). However, among those individuals who had ED experience the utilisation rate was lower (RR = 0.96) (Table 3).

The interaction between the origin area and the time-period suggests that the effect of time on the propensity of being an ED user was stronger for migrants from Central-Eastern Europe and weaker for those from Africa and Asia. The decrease in the ED utilisation rate did not, meanwhile, change by origin area (Table 3A in the Additional file).

When migrant status was classified as Italians, HMPC as a whole group, and HDC, the propensity to use the ED was always lower for migrants (HDC: OR = 0.23; HMPC: OR = 0.53), and a lower (HDC: RR = 0.65) or similar (HMPC: RR = 1.00) ED utilisation rate was found compared to Italians (Table 5A in the Additional file).

### Results by selected causes

Regardless of cause, all migrant subgroups were less likely to be ED users than non-migrants (zero-part model). The count-part model displays that for cardiovascular diseases, only migrants from HDC registered a lower utilisation rate (RR = 0.77). As to mental disorders, lower ED utilisation rates belonged to Latin Americans, Asians, and Central-Eastern Europeans, while for injuries all migrant subgroups had lower rates than Italians.

Looking at the relation between ED use and time-period, as compared to the pre-2008, in the post-2008 time-period we detected both an increase in the likelihood of being an ED user, and a decrease in ED utilisation rates, for the total population.

The interaction between origin area and time-period suggests that, over time, the likelihood of being ED users was stronger among Latin Americans and Central-Eastern Europeans for cardiovascular diseases; among Central-Eastern Europeans for mental disorders; among Latin Americans and Central-Eastern Europeans for injuries; whereas it was weaker among Africans and Asians. Concerning the count-part model, in the post-2008 period, ED utilisation rates did not change by origin area for cardiovascular diseases and mental disorders, while for injuries the decrease in the ED rates was weaker for Latin-Americans, and stronger for Asians (Table 4A in the Additional file).

When migrant status was classified as Italians, HMPC, and HDC, migrants again showed a lower propensity of having ED experience for all selected causes and lower ED utilisation rates, except for cardiovascular diseases.

As compared to the pre-2008, in the post-2008 time-period we registered a higher propensity of being an ED user and a lower ED utilisation rate, for the whole population. Over time, the propensity for using the ED was stronger among HMPC migrants for cardiovascular diseases (OR = 1.24) and mental disorders (OR = 1.17). Meanwhile, no differences were detected in ED utilisation rates (Table 6A in the Additional file).

## Discussion

This is the first study which analysed disparities in emergency department use between migrants and Italians in Rome by linking the demographic information provided by the Rome Municipal Register with ED data and using an eleven-year longitudinal study of individuals. The first aim of this study was to investigate the association between migrant status and ED use from 2005 to 2015; the second aim was to examine changes in the ED use by comparing the pre-2008 and the post-2008 time-period for all causes and for selected causes. The current study suggests that the Hurdle model, based on the assumption of two different processes respectively for the contact and the frequency decision, allows to provide a complete picture of the ED use. Indeed, it was possible to assess and provide insight into how relevant migrant status is in determining both the decision to contact and how frequently to use the emergency care service, which is often of substantive policy interest.

As regards the former aim, the analyses showed that migrants have a lower probability to contact the ED than Italians. This result is in line with other international studies [43–46]. Following the international literature, and being cautious in the interpretation of data, this finding might be due to a better migrants’ health status and migrants’ healthier lifestyles, known in literature as the healthy migrant effect [21], which have also been proven in the Italian context [50, 51, 68, 69]. Nevertheless, looking at the frequency of use among those who accessed the ED, the analysis displays that migrants registered a different utilisation rate, which varies according to the origin area. Those from HDC, Asia and Central-Eastern Europe show lower ED use than Italians. Conversely, results report higher utilisation rates among non-European migrants, particularly those from the “global South” (Africans and Latin-Americans) than non-migrants. The higher frequency in the ED use by some migrant subgroups compared to Italians is in line with previous findings [34–37]. There might be different explanations of such pattern. Emergency services are easier to access for migrants and they provide an immediate solution to their healthcare needs by reducing linguistic, bureaucratic, economic, cultural and organizational barriers that migrants may face [21, 24, 40, 59] when trying to access primary (general practitioners) and secondary (specialist visits) care. Thus, they may tend to use the ED as a first choice [36, 41, 70]. The ED use can also be influenced by differences in the health status and urgent medical conditions between migrants and non-migrants. Furthermore, a study conducted in eight Italian regions, which investigated the use of the healthcare services by migrants, found higher ED contact percentages and utilisation rates for cases classified as non-urgent among migrants compared to Italians [71]. Similarly, Zinelli et al. (2014) [58] and Buja et al. (2014) [72] found the same results for different Italian contexts. Although we did not perform an analysis taking into account the triage classification which allows to discriminate between urgent and non-urgent cases, the studies abovementioned suggests that our finding might also reflect an inappropriate use of the emergency care, i.e., the excess of ED use observed among specific migrant subgroups might be driven by non-urgent cases.

With respect to the second aim, which refers to changes in ED use over time, by comparing the pre-2008 and the post-2008 time-period ED use, we found, for the whole population, an increase in the probability to contact the ED and a decrease in the frequency of use the emergency service among those who accessed at least once, with some differences among migrant subgroups, regardless of cause.

As argued by Hughes and Khaliq (2014), the increase in the likelihood to contact the ED might be related to the economic situation and to the implementation and adoption of austerity policies [73]. These may have reduced healthcare assistance, either because of the limited availability of health services or because of increased costs [74] during the Great Recession. In a study conducted in Greece, Kyriopoulos et al. (2014) [75] found that economic downturns and times of austerity have negative impacts on individuals’ access to healthcare services, by increasing economic/financial barriers which can be mainly attributable to income decrease and unemployment. In this regard, the European Commission (2016) [76] affirmed that financial barriers are the largest single driver of unmet need for healthcare in the European Union. In the Italian context, on the one hand, the economic recession restrains both public and private health sector expenditure, making it difficult to meet the health needs and expectations of the population. On the other hand, the high national debt stock pushes to improve public finances to avoid default, by forcing singular public spending cuts. In a recent Italian study, Busetta et al. (2018) [77] claimed that many individuals have experienced barriers in access medical care due to unaffordability and unavailability of health services. They also affirmed that the situation is especially challenging for migrants. In this regard, an interaction effect between the decision to contact the ED and migrant subgroups suggests that such increase was stronger especially among Latin Americans and Central-Eastern Europeans. On the one hand, this finding might be due to access barriers [24, 29, 72] when seeking primary and secondary care that may grow during economic downturns, mainly economic barriers, which can be affected by fiscal policy, beyond the health system. On the other hand, this result may support our second hypothesis that migrants’ health status worsens over time. Their age, acculturation, economic downturns, changes in lifestyles, and difficult living and working conditions in the host country, might affect their healthcare need profile, thus a decline in the health advantage may occur, known in the literature as the exhausted migrant effect [23, 44, 78]. This finding is consistent with other studies which suggest that new living conditions and a longer length of stay in the host country negatively influences migrants’ health status [79–82].

Conversely, by analysing the frequency of ED use over time, compared to the pre-2008, in the post-2008 time-period we observed a decrease in the ED utilisation rate for the whole population. According to some studies which investigated the relationships between business cycles and health or mortality, this finding could be due to the correction of unhealthy habits, and thus it might be linked to a general improvement in health status [83, 84]. Furthermore, such pattern can also be interpreted by referring to the so-called *Thomas effect* [85], generally mentioned in mortality analyses, whereby access to ED decreases with economic contractions, instead of being countercyclical, as expected [86, 87]. In the Italian context, this kind of a decrease is also related to the reorganization of health services to reduce inappropriate emergency care use (Law 296/2006 art 1 paragraph 796) [88], by introducing co-payments which were added to existing tariffs, placing a significant additional burden on patients [89].

It should finally be mentioned that, although the Arab Spring in 2011 and the humanitarian emergency in 2014–2015 marked the migration phenomenon in Italy, the figures on refugees, asylum seekers and holders of humanitarian protection show that Italy’s main role is that of a country of transit for the migration flows toward the European Union [3]. Indeed, in the period under examination, the peak in the number of these vulnerable populations was reached only in 2015. Despite in our study we cannot distinguish refugees or asylum seekers, based on the above considerations we surmise that the effect of the ED use by such individuals on our findings is negligible.

The main limitation of the study can be ascribed to the administrative nature of the data, which do not include important confounders or relevant risk factors changing over time, which can influence the ED utilisation. First, we could not account for socioeconomic status: being out of the labour force or having a low socio-economic status increases the likelihood of using medical services [90, 91]. In addition, length of stay in the host country may be another important confounder, which may influence migrants’ healthcare utilisation patterns [80–82]. Another limitation is related to potential exposure misclassification in relation to changes of citizenship over time. This, however, is likely to be negligible because of the low rate of citizenship acquisition registered in Italy (1.1% in 2005 and 2.6% in 2015) [92]. Finally, a source of bias might be the lack of information about ED contacts on the part of Rome residents in other Italian regions. The percentage of residents in Rome who access emergency care in other regions is likely, though, to be very low because the city is in the centre of Lazio, far away from the regional borders.

The use of data from a subgroup of the migrant population resident in Italy does not limit the external validity of our results, although this validity is probably restricted to metropolitan areas, which are characterised by similar job opportunities, services and migrant networks (e.g., Milan, Venice, Turin, Florence, etc.). As regards the composition of migrant flows, it should also be stressed that the distribution of the migrant population in Rome and in Italy is quite similar. In both cases Romanians are the first community, and about 50% of migrants come from Central-Eastern Europe [1]. Moreover, the healthcare provision in the city of Rome includes all the variety of hospital profiles (university, private and public hospitals), reflecting the complexity of the national healthcare services.

Despite these limitations, our study takes a step forward in a better understanding of the ED use among migrants. The study is in agreement with several studies conducted in Denmark [40], Spain [43, 45], USA [44], and Canada [46] which found that migrant subgroups are less likely to use EDs than non-migrants, and tend to have a better health status and lower mortality rates than non-migrants. However, those works analyse either the decision to contact the ED (using logistic regressions) or the frequency of use (using count data models), and none of them consider the two aspects simultaneously. Furthermore, most of those studies tend to define migrants as a unique group, without taking into account the specificities of migrants’ countries of origin. Moreover, most of the aforementioned studies use cross-sectional data and do not cover more than four to five years.

The added value of our study is to distinguish among the decision to contact the ED and the frequency of use, and also to highlight on the importance of looking at each migrant subgroups in relation to their peculiarities according to geographical origin, instead of grouping migrants into a broad category. In particular, by applying the Hurdle model we analysed both the probability to contact the ED, and the frequency of multiple attendances. Indeed, what clearly emerged from our findings is that among those migrants who accessed the emergency service, some specific migrant subgroups registered an over-use of the ED compared to non-migrants, which is consistent with previous findings [31, 34–37]. Finally, the use of a longitudinal design allows to compute denominators accurately, based on person-time, and to estimate unbiased utilisation rates.

## Conclusions

In Italy, the migrant population accounts for 8.7% of the total population. However, knowledge about migrants’ health service use is still incomplete. Studying the ED use is relevant because it reflects both the need for urgent assistance, and it is also an indicator of accessibility and quality of care. It is not a health indicator per se, but it is strictly related to health indicators and it may vary during periods of crisis [41]. Moreover, the ED can capture health needs and behaviour in real time, in contrast to other indicators coming from health surveys which may take longer to detect changes. Our study follows and pairs with the results of two recent longitudinal studies conducted in Italy between 2001 and 2013, showing that overall hospitalization is lower among migrants compared to Italians [93], and that migrants are at higher risk of undergoing hospital admissions that could be avoided through appropriate outpatient care, suggesting they may experience lower primary healthcare accessibility than the non-migrant population [94].

Most countries grant full equality of treatment to third country nationals after awarding them permanent residence status. So, is access to health care still an issue? Data on this topic are relatively sparse, but several studies suggest migrants do experience unequal access to health care.

Our findings draw attention to contemporary debate on healthcare and international migrants, two topics discussed worldwide, and point to the need for interventions in order to reduce access barriers to health services, especially among specific groups of migrants. In the European context, Italy is progressive in the migrant health protection. The Italian National Health Service is inclusive and guarantees that regular migrants have the same rights as Italian citizens. In addition, it admits opportunities for health protection and assistance for irregular migrants. Nonetheless, even if in Italy equity remains a fundamental principle of the health system, our study highlights disparities in the use of the emergency service. The persistence of differences in the healthcare utilisation between migrants and non-migrants, and within the whole migrant group depending on the geographical area of origin, deserves further research. Our study shows that administrative data provide useful information to monitor migrants’ healthcare use. We found that Africans and Latin Americans have higher ED utilisation rates, while the other migrant groups have lower ED utilisation rates. Interpretation of those results should consider the heterogeneity that characterizes the migrant population in relation to many underlying factors (culture, integration in the host country, health literacy, job and income, demographic structure, social cohesion and support of their own community). Therefore, interventions to tackle those health inequalities should go beyond improving health services to include policy measures addressing the underlying factors, adopting a Health in All Policies perspective [95]. It is also relevant to better understand whether such utilisation differences are related to migrants’ health status, to health needs, or to access barriers that especially some groups of migrants must face in seeking care. From a policy perspective and due to the complexity of the issues and the period analysed in this study, which was characterized by changes in the composition of migration flows, the economic downturn, and healthcare spending cuts, the study suggests the importance to develop further researches, also focusing on specific groups of migrants and on the causes and diagnoses underlying the use of health services, to achieve a more comprehensive understanding of the factors that determine the differences observed in the emergency service utilisation patterns of migrants and non-migrants.

## Supplementary information

**Additional file 1.** Supplementary tables.

## Data Availability

The datasets generated and/or analysed during the current study are not publicly available due to stringent legal restrictions regarding privacy policy on personal information in Italy (national legislative decree on privacy policy n. 196/30 June 2003). In addition, due to security aspects they can be analysed only in a safe place. Researchers may contact the corresponding author for questions concerning the data which are however available from the authors upon reasonable request and with permission of the Italian Data Protection Authority.

## Abbreviations

ED: Emergency Department
SSN: National Health Service
WHO: World Health Organization
STP: Temporary Foreigner
IN-liMeS: Italian Network of Longitudinal Metropolitan studies
HIS-EC: Health Information System on Emergency Care
ICD-9-CM: International Classification of Diseases, 9th revision – Clinical Modification
HMPC: High Migratory Pressure Countries
HDC: Highly Developed Countries
SUR: Direct age-standardized utilisation rates
RR: Relative Risk
OR: Odds Ratio
CI: Confidence Interval

## Footnotes

1 We conducted a sensitivity analysis including individuals who were excluded from the computations (1.1%), assuming that their use of the ED was the same as observed individuals. Results remain virtually unchanged.
