# Supplementary material for: Disparities in emergency department use between Italians and migrants residing in Rome, Italy: the Rome Dynamic Longitudinal Study from 2005 to 2015
Source: BMC Public Health. 2020 Oct 15;20:1548. doi: 10.1186/s12889-020-09280-6 (PMC7559990; doi:10.1186/s12889-020-09280-6)
Supplement: Supplementary file 1 — Additional file 1. Supplementary tables. [file 12889_2020_9280_MOESM1_ESM.docx]

**ADDITIONAL FILE**

**Table 3A. Hurdle model: all-cause ED contacts by origin area and time-period, 2005-2015**

|  | **Zero-part model** | | |  | **Count-part model** | | |
| --- | --- | --- | --- | --- | --- | --- | --- |
|  | **OR** |  | **95% CI** |  | **RR** |  | **95% CI** |
| **Origin area** |  |  |  |  |  |  |  |
| Italy^a^ |  |  |  |  |  |  |  |
| HDC | 0.23 | *** | (0.22-0.24) |  | 0.65 | *** | (0.61-0.70) |
| Africa | 0.52 | *** | (0.50-0.53) |  | 1.46 | *** | (1.40-1.52) |
| Latin-America | 0.72 | *** | (0.70-0.73) |  | 1.04 | * | (1.00-1.08) |
| Asia | 0.43 | *** | (0.43-0.44) |  | 0.80 | *** | (0.78-0.83) |
| Eastern-Europe | 0.55 | *** | (0.54-0.55) |  | 0.96 | *** | (0.93-0.98) |
| **Gender** |  |  |  |  |  |  |  |
| Man^a^ |  |  |  |  |  |  |  |
| Woman vs Man | 1.02 | *** | (1.01-1.02) |  | 1.06 | *** | (1.05-1.06) |
| **Age group** |  |  |  |  |  |  |  |
| 25-34^a^ |  |  |  |  |  |  |  |
| 35-44 | 1.35 | *** | (1.34-1.36) |  | 0.86 | *** | (0.85-0.86) |
| 45-54 | 1.05 | *** | (1.04-1.05) |  | 0.69 | *** | (0.68-0.69) |
| 55-64 | 0.79 | *** | (0.78-0.79) |  | 0.67 | *** | (0.66-0.68) |
| **Time-period** |  |  |  |  |  |  |  |
| Pre-2008^a^ |  |  |  |  |  |  |  |
| Post-2008 | 1.34 | *** | (1.34-1.35) |  | 0.96 | *** | (0.96-0.97) |
| **Origin area * Time-period** | |  |  |  |  |  |  |
| HDC Post-2008 | 0.98 |  | (0.93-1.02) |  | 0.94 |  | (0.87-1.02) |
| Africa Post-2008 | 0.80 | *** | (0.77-0.82) |  | 1.00 |  | (0.96-1.05) |
| Latin-America Post-2008 | 0.99 |  | (0.96-1.02) |  | 1.01 |  | (0.97-1.06) |
| Asia Post-2008 | 0.93 | *** | (0.91-0.95) |  | 0.99 |  | (0.96-1.03) |
| Eastern-Europe Post-2008 | 1.21 | *** | (1.19-1.23) |  | 0.99 |  | (0.96-1.01) |
| *N observations* | *3772584* | |  |  | *1735368* | |  |

^a^ Reference category

Note: The asterisks indicate significance *p < 0.05, **p < 0.01, ***p < 0.001.

**Table 4A. Hurdle Model: CVDs, mental disorders, injuries ED contacts by origin area and time-period, 2005-2015**

|  | **CVDs** | | | | | |  | **Mental disorders** | | | | | |  | **Injuries** | | | | | |
| --- | --- | --- | --- | --- | --- | --- | --- | --- | --- | --- | --- | --- | --- | --- | --- | --- | --- | --- | --- | --- |
|  | **Zero-part model** | | | **Count-part model** | | |  | **Zero-part model** | | | **Count-part model** | | |  | **Zero-part model** | | | **Count-part model** | | |
|  | **OR** |  | **95% CI** | **RR** |  | **95% CI** |  | **OR** |  | **95% CI** | **RR** |  | **95% CI** |  | **OR** |  | **95% CI** | **RR** |  | **95% CI** |
| **Origin area** |  |  |  |  |  |  |  |  |  |  |  |  |  |  |  |  |  |  |  |  |
| Italy^a^ |  |  |  |  |  |  |  |  |  |  |  |  |  |  |  |  |  |  |  |  |
| HDC | 0.17 | *** | (0.14-0.22) | 0.77 | *** | (0.37-1.62) |  | 0.22 | *** | (0.17-0.28) | 0.71 |  | (0.39-1.29) |  | 0.25 | *** | (0.23-0.26) | 0.69 | *** | (0.62-0.77) |
| Africa | 0.47 | *** | (0.40-0.54) | 1.09 |  | (0.75-1.58) |  | 0.47 | *** | (0.41-0.54) | 0.78 |  | (0.56-1.09) |  | 0.42 | *** | (0.40-0.43) | 0.89 | *** | (0.84-0.95) |
| Latin-America | 0.35 | *** | (0.29-0.42) | 0.97 |  | (0.58-1.62) |  | 0.54 | *** | (0.47-0.62) | 0.41 | *** | (0.28-0.61) |  | 0.58 | *** | (0.56-0.60) | 0.85 | *** | (0.80-0.90) |
| Asia | 0.59 | *** | (0.54-0.65) | 1.05 |  | (0.83-1.34) |  | 0.24 | *** | (0.21-0.28) | 0.63 | ** | (0.45-0.88) |  | 0.31 | *** | (0.30-0.32) | 0.61 | *** | (0.58-0.65) |
| Eastern-Europe | 0.49 | *** | (0.45-0.54) | 1.19 |  | (0.94-1.51) |  | 0.49 | *** | (0.45-0.53) | 0.62 | *** | (0.50-0.77) |  | 0.47 | *** | (0.46-0.48) | 0.85 | *** | (0.82-0.89) |
| **Gender** |  |  |  |  |  |  |  |  |  |  |  |  |  |  |  |  |  |  |  |  |
| Man^a^ |  |  |  |  |  |  |  |  |  |  |  |  |  |  |  |  |  |  |  |  |
| Woman | 0.60 | *** | (0.59-0.61) | 0.86 | *** | (0.83-0.89) |  | 1.13 | *** | (1.12-1.15) | 0.76 | *** | (0.73-0.79) |  | 0.72 | *** | (0.71-0.72) | 0.72 | *** | (0.71-0.72) |
| **Age_group** |  |  |  |  |  |  |  |  |  |  |  |  |  |  |  |  |  |  |  |  |
| 25-34^a^ |  |  |  |  |  |  |  |  |  |  |  |  |  |  |  |  |  |  |  |  |
| 35-44 | 2.67 | *** | (2.60-2.75) | 1.37 | *** | (1.18-1.37) |  | 1.05 | *** | (1.03-1.07) | 1.13 | *** | (1.08-1.18) |  | 0.98 | *** | (0.98-0.99) | 0.89 | *** | (0.88-0.90) |
| 45-54 | 5.64 | *** | (5.49-5.79) | 1.79 | *** | (1.55-1.79) |  | 0.91 | *** | (0.89-0.93) | 1.08 | ** | (1.03-1.13) |  | 0.91 | *** | (0.90-0.92) | 0.83 | *** | (0.83-0.84) |
| 55-64 | 6.69 | *** | (6.52-6.87) | 2.39 | *** | (2.07-2.39) |  | 0.51 | *** | (0.49-0.52) | 0.94 | * | (0.88-1.00) |  | 0.56 | *** | (0.55-0.56) | 0.77 | *** | (0.76-0.77) |
| **Time-period** |  |  |  |  |  |  |  |  |  |  |  |  |  |  |  |  |  |  |  |  |
| Pre-2008^a^ |  |  |  |  |  |  |  |  |  |  |  |  |  |  |  |  |  |  |  |  |
| Post-2008 | 1.60 | *** | (1.57-1.62) | 0.88 | *** | (0.85-0.91) |  | 1.31 | *** | (1.28-1.33) | 0.91 | *** | (0.88-0.95) |  | 1.21 | *** | (1.20-1.22) | 0.82 | *** | (0.81-0.83) |
| **Origin area * Time-period** |  |  |  |  |  |  |  |  |  |  |  |  |  |  |  |  |  |  |  |  |
| HDC Post-2008 | 1.23 |  | (0.92-1.65) | 1.07 |  | (0.46-2.49) |  | 0.94 |  | (0.70-1.27) | 1.40 |  | (0.69-2.85) |  | 0.99 |  | (0.93-1.07) | 0.90 |  | (0.79-1.04) |
| Africa Post-2008 | 1.14 |  | (0.96-1.35) | 0.86 |  | (0.56-1.31) |  | 0.93 |  | (0.79-1.11) | 1.08 |  | (0.73-1.60) |  | 0.81 | *** | (0.78-0.85) | 0.99 |  | (0.93-1.06) |
| Latin-America Post-2008 | 1.26 | * | (1.02-1.56) | 1.01 |  | (0.57-1.80) |  | 1.11 |  | (0.94-1.31) | 1.11 |  | (0.70-1.74) |  | 1.05 | * | (1.00-1.10) | 1.08 | * | (1.01-1.16) |
| Asia Post-2008 | 1.09 |  | (0.98-1.21) | 0.95 |  | (0.72-1.24) |  | 0.95 |  | (0.81-1.12) | 0.94 |  | (0.63-1.40) |  | 0.91 | *** | (0.88-0.94) | 0.93 | * | (0.87-0.99) |
| Eastern-Europe Post-2008 | 1.41 | *** | (1.27-1.56) | 0.82 |  | (0.63-1.07) |  | 1.42 | *** | (1.29-1.56) | 1.10 |  | (0.87-1.39) |  | 1.20 | *** | (1.17-1.23) | 1.00 |  | (0.96-1.04) |
| *N observations* | *3772584* | |  | *84610* | |  |  | *3772584* | |  | *57820* | |  |  | *3772584* | |  | *882930* | |  |

^a^ Reference category

Note: The asterisks indicate significance *p < 0.05, **p < 0.01, ***p < 0.001.

**Table 5A. Hurdle model: all-cause ED contacts by migrant status and time-period, 2005-2015**

|  | **Zero-part model** | | |  | **Count-part model** | | |
| --- | --- | --- | --- | --- | --- | --- | --- |
|  | **OR** | | **95% CI** |  | **RR** | | **95% CI** |
| **Origin area** |  |  |  |  |  |  |  |
| Italy^a^ |  |  |  |  |  |  |  |
| HDC | 0.23 | *** | (0.22-0.24) |  | 0.65 | *** | (0.61-0.70) |
| HMPC | 0.53 | *** | (0.52-0.53) |  | 1.00 |  | (0.98-1.02) |
| **Gender** |  |  |  |  |  |  |  |
| Man^a^ |  |  |  |  |  |  |  |
| Woman vs Man | 1.03 | *** | (1.03-1.04) |  | 1.06 | *** | (1.05-1.06) |
| **Age group** |  |  |  |  |  |  |  |
| 25-34^a^ |  |  |  |  |  |  |  |
| 35-44 | 1.35 | *** | (1.34-1.36) |  | 0.86 | *** | (0.85-0.86) |
| 45-54 | 1.05 | *** | (1.04-1.06) |  | 0.69 | *** | (0.68-0.70) |
| 55-64 | 0.79 | *** | (0.79-0.80) |  | 0.67 | *** | (0.66-0.68) |
| **Time-period** |  |  |  |  |  |  |  |
| Pre-2008^a^ |  |  |  |  |  |  |  |
| Post-2008 | 1.34 | *** | (1.34-1.35) |  | 0.96 | *** | (0.96-0.97) |
| **Origin area * Time-period** |  |  |  |  |  |  |  |
| HDC Post-2008 | 0.98 |  | (0.93-1.02) |  | 0.94 |  | (0.87-1.02) |
| HMPC Post-2008 | 1.01 |  | (1.00-1.02) |  | 0.98 | * | (0.96-0.99) |
| *N observations* | *3772584* | |  |  | *1735368* | |  |

^a^ Reference category

Zero-part model reports odds ratios for the outcome variable indicating persons without (Y = 0) or with Emergency Department experience (Y = 1, where all values larger than 0 are censored, which means, are fixed at 1), while the Count-part model, which reports rate ratios, models the number of Emergency Department experiences for those with Emergency Department experiences (for those with Y >0).

Note: The asterisks indicate significance *p < 0.05, **p < 0.01, ***p < 0.001.

**Table 6A. Hurdle Model: CVDs, mental disorders, injuries ED contacts by migrant status and time-period, 2005-2015**

|  | **CVDs** | | | | | |  | **Mental disorders** | | | | | |  | **Injuries** | | | | | |
| --- | --- | --- | --- | --- | --- | --- | --- | --- | --- | --- | --- | --- | --- | --- | --- | --- | --- | --- | --- | --- |
|  | **Zero-part model** | | | **Count-part model** | | |  | **Zero-part model** | | | **Count-part model** | | |  | **Zero-part model** | | | **Count-part model** | | |
|  | **OR** |  | **95% CI** | **RR** |  | **95% CI** |  | **OR** |  | **95% CI** | **RR** |  | **95% CI** |  | **OR** |  | **95% CI** | **RR** |  | **95% CI** |
| **Origin area** |  |  |  |  |  |  |  |  |  |  |  |  |  |  |  |  |  |  |  |  |
| Italy^a^ |  |  |  |  |  |  |  |  |  |  |  |  |  |  |  |  |  |  |  |  |
| HDC | 0.17 | *** | (0.14-0.22) | 0.78 |  | (0.37-1.63) |  | 0.22 | *** | (0.17-0.28) | 0.71 |  | (0.39-1.29) |  | 0.25 | *** | (0.23-0.26) | 0.69 | *** | (0.62-0.77) |
| HMPC | 0.50 | *** | (0.47-0.53) | 1.10 |  | (0.95-1.28) |  | 0.42 | *** | (0.40-0.45) | 0.61 | *** | (0.53-0.71) |  | 0.43 | *** | (0.42-0.43) | 0.80 | *** | (0.78-0.82) |
| **Gender** |  |  |  |  |  |  |  |  |  |  |  |  |  |  |  |  |  |  |  |  |
| Man^a^ |  |  |  |  |  |  |  |  |  |  |  |  |  |  |  |  |  |  |  |  |
| Woman | 0.60 | *** | (0.59-0.61) | 0.86 | *** | (0.83-0.89) |  | 1.15 | *** | (1.13-1.17) | 0.76 | *** | (0.73-0.79) |  | 0.72 | *** | (0.72-0.73) | 0.72 | *** | (0.71-0.72) |
| **Age_group** |  |  |  |  |  |  |  |  |  |  |  |  |  |  |  |  |  |  |  |  |
| 25-34^a^ |  |  |  |  |  |  |  |  |  |  |  |  |  |  |  |  |  |  |  |  |
| 35-44 | 2.67 | *** | (2.60-2.75) | 1.27 | *** | (1.18-1.37) |  | 1.05 | *** | (1.03-1.07) | 1.13 | *** | (1.08-1.18) |  | 0.98 | *** | (0.98-0.99) | 0.90 | *** | (0.88-0.90) |
| 45-54 | 5.64 | *** | (5.49-5.78) | 1.67 | *** | (1.55-1.79) |  | 0.91 | *** | (0.89-0.93) | 1.08 | ** | (1.03-1.14) |  | 0.91 | *** | (0.91-0.92) | 0.83 | *** | (0.83-0.84) |
| 55-64 | 6.69 | *** | (6.52-6.87) | 2.22 | *** | (2.07-2.39) |  | 0.51 | *** | (0.49-0.52) | 0.93 | * | (0.88-1.00) |  | 0.56 | *** | (0.55-0.56) | 0.77 | *** | (0.76-0.77) |
| **Time-period** |  |  |  |  |  |  |  |  |  |  |  |  |  |  |  |  |  |  |  |  |
| Pre-2008^a^ |  |  |  |  |  |  |  |  |  |  |  |  |  |  |  |  |  |  |  |  |
| Post-2008 | 1.60 | *** | (1.57-1.62) | 0.88 | *** | (0.85-0.91) |  | 1.31 | *** | (1.28-1.33) | 0.91 | *** | (0.88-0.95) |  | 1.21 | *** | (1.20-1.22) | 0.82 | *** | (0.81-0.83) |
| **Origin area * Time-period** |  |  |  |  |  |  |  |  |  |  |  |  |  |  |  |  |  |  |  |  |
| HDC Post-2008 | 1.23 |  | (0.92-1.65) | 1.07 |  | (0.46-2.48) |  | 0.94 |  | (0.70-1.28) | 1.40 |  | (0.69-2.84) |  | 0.99 |  | (0.93-1.07) | 0.90 |  | (0.79-1.04) |
| HMPC Post-2008 | 1.24 | *** | (1.16-1.32) | 0.89 |  | (0.75-1.05) |  | 1.17 | *** | (1.09-1.26) | 1.08 |  | (0.91-1.27) |  | 1.01 |  | (1.00-1.03) | 0.99 |  | (0.97-1.03) |
| N observations | *3772584* | |  | *84610* | |  |  | *3772584* | |  | *57820* | |  |  | *3772584* | |  | *882930* | |  |

^a^ Reference category

Zero-part model reports odds ratios for the outcome variable indicating persons without (Y = 0) or with Emergency Department experience (Y = 1, where all values larger than 0 are censored, which means, are fixed at 1), while the Count-part model, which reports rate ratios, models the number of Emergency Department experiences for those with Emergency Department experiences (for those with Y >0).

Note: The asterisks indicate significance *p < 0.05, **p < 0.01, ***p < 0.001.
